# Combined analysis of single-cell and bulk RNA sequencing reveals the expression patterns of circadian rhythm disruption in the immune microenvironment of Alzheimer’s disease

**DOI:** 10.3389/fimmu.2023.1182307

**Published:** 2023-05-12

**Authors:** Huiling He, Yingxia Yang, Lingxing Wang, Zeming Guo, Lichao Ye, Wanjiong Ou-Yang, Meili Yang

**Affiliations:** Department of Neurology, The Second Attached Hospital of Fujian Medical University, Quanzhou, Fujian, China

**Keywords:** single-cell, circadian rhythm, immune microenvironment, Alzheimer’s disease, machine learning

## Abstract

**Background:**

Circadian rhythm disruption (CRD) represents a critical contributor to the pathogenesis of Alzheimer’s disease (AD). Nonetheless, how CRD functions within the AD immune microenvironment remains to be illustrated.

**Methods:**

Circadian rhythm score (CRscore) was utilized to quantify the microenvironment status of circadian disruption in a single-cell RNA sequencing dataset derived from AD. Bulk transcriptome datasets from public repository were employed to validate the effectiveness and robustness of CRscore. A machine learning-based integrative model was applied for constructing a characteristic CRD signature, and RT-PCR analysis was employed to validate their expression levels.

**Results:**

We depicted the heterogeneity in B cells, CD4^+^ T cells, and CD8^+^ T cells based on the CRscore. Furthermore, we discovered that CRD might be strongly linked to the immunological and biological features of AD, as well as the pseudotime trajectories of major immune cell subtypes. Additionally, cell–cell interactions revealed that CRD was critical in the alternation of ligand-receptor pairs. Bulk sequencing analysis indicated that the CRscore was found to be a reliable predictive biomarker in AD patients. The characteristic CRD signature, which included 9 circadian‐related genes (CRGs), was an independent risk factor that accurately predicted the onset of AD. Meanwhile, abnormal expression of several characteristic CRGs, including GLRX, MEF2C, PSMA5, NR4A1, SEC61G, RGS1, and CEBPB, was detected in neurons treated with Aβ1-42 oligomer.

**Conclusion:**

Our study revealed CRD-based cell subtypes in the AD microenvironment at single-cell level and proposed a robust and promising CRD signature for AD diagnosis. A deeper knowledge of these mechanisms may provide novel possibilities for incorporating “circadian rhythm-based anti-dementia therapies” into the treatment protocols of individualized medicine.

## Introduction

Alzheimer’s disease (AD) is the most prevalent age-related neurodegenerative disease in the world, accounting for approximately 70% of the 50 million individuals worldwide with dementia, with about 10 million new cases per year, or nearly 20 new cases every minute (1). It is generally accepted that the amyloid plaques and hyperphosphorylated tau protein observed in the brains are recognized as the hallmarks of AD, thus leading to the progressive memory and cognitive impairment (2). Although several FDA-approved drugs have been developed in recent decades to slow the progression of Alzheimer’s disease, the heterogeneity and complicated pathophysiological mechanisms underlying Alzheimer’s disease limit their efficacy (3, 4). Therefore, with an aging population, a better knowledge of AD pathophysiology is critical for the development of effective therapeutics.

Circadian rhythm (CR) is a physiologic cycle of approximately 24 hours that has been characterized as an evolutionary molecular mechanism to coordinate multiple physiological processes, such as energy metabolism and self-sustenance, via an established circadian clock (5). A growing body of evidence suggests that disruption of the circadian rhythm plays a critical role in cognitive impairment and facilitates the development and progression of Alzheimer’s disease (AD). In a genetically engineered AD mouse model, for example, abnormal expression of circadian rhythm-related genes such as BMAL1, CLOCK, and PER was observed, implying a possible link between altered circadian rhythm, Aβ pathology, and tauopathy (6). Furthermore, circadian rhythm disruption (CRD) has been demonstrated to promote oxidative damage and the development of AD by interacting with Aβ42 and disrupting redox homeostasis (7, 8). Furthermore, PRX, a non-transcriptional rhythm regulator, is reduced in AD and is associated with memory impairment and a poor prognosis (9). It is worth noting that key circadian components either directly or indirectly regulate the expression of various peripheral circadian genes, which appear to be closely linked to the progression of neurodegenerative disease (10, 11). Furthermore, dysregulation of circadian rhythm-related genes complicates AD pathogenesis, leading to a poor prognosis and failure of AD therapies (12). However, to our knowledge, CRD‐based AD pathogenesis has been investigated mainly using data from the samples produced from genetically engineered *in vivo* or *in vitro* models (6, 8). Although these studies have contributed to a better understanding of the interplay between disrupted circadian rhythms and AD progression, they are mainly based on the average state of large tissues and overall cells under different genetics and environments, leaving out the consideration of the diversity of biological processes within individual cells. It is worth noting that immune cells living in the AD microenvironment including T cells, B cells, macrophages, monocytes, and others, play a pivotal role in the initiation and progression of AD (13, 14). Furthermore, it has been reported that the circadian rhythm regulates multiple aspects of immunity through a complex reorganization of cellular connections, thereby preventing cytokine-mediated inflammatory responses during the development of chronic inflammation and carcinogenesis (15). Furthermore, overexpression of BMAL1 in malignant tumors has been shown to be positively related to T cell infiltration and activation (16). However, whether circadian rhythm drives AD progression in the complex immune microenvironment, as well as the potential mechanism underlying the interaction between circadian gene dysregulation and AD progression at single-cell resolution, remain unknown and require further research.

In this study, we exhibited the CRD state in the AD immune microenvironment at the at single‐cell level based on the transcriptomic profiles of circadian‐related genes (CRGs). What’s more, we investigated the impact of the CRscore on the main immune cells, including B cells, CD4^+^ T cells, and CD8^+^ T cells, based on the single-cell sequencing data derived from the AD sample. Our results revealed that different levels of CRscore in each immune cell type subpopulation were closely related to distinct immune characteristics, intercellular communications, metabolic pathways, and transcription characteristics. Importantly, bulk transcriptomic analysis demonstrated that a lower CRscore was significantly correlated with AD pathogenesis and progression. Furthermore, characteristic CRGs selected by multiple integrative machine learning algorithms were utilized to construct a robust model for predicting the onset of AD. In conclusion, we clarified the close relationship between CRD and AD heterogeneity by combining bulk RNA sequencing and single-cell analysis, and pharmacological modulation targeting circadian genes may be a promising therapeutic strategy for AD combination therapy.

## Methods

### Data acquisition and processing

The single-cell mRNA sequence (scRNA-seq) matrix of GEO dataset GSE181279, including 2 normal and 3 AD peripheral blood samples, was downloaded from the Gene Expression Omnibus (GEO, www.ncbi.nlm.nih.gov/geo/) database and further reanalyzed in the current study. Using the R software’s Seurat package, we generated Seurat objects for all specific cell types comprising the scRNA-seq gene expression matrix. Then, the Seurat package’s IntegrateData was utilized to perform batch elimination and sample integration. Using FindVariableFeatures from the Seurat package, the top 2000 genes with highly variable expression were identified. In addition, we chose cells that expressed between 200 and 4000 genes, and less than 20% mitochondrial genes. The remaining scRNA-seq data were normalized and scaled using the NormalizeData and ScaleData functions from the Seurat package for additional analysis. Using RunPCA of the Seurat package, the number of principal components (PC) was estimated, and the uniform manifold approximation and projection (UMAP) reduction analysis was employed to summarize the top principal components. The cell types were manually annotated using known gene markers based on previous publications. Using the Idents and DimPlot tools, general cell types or subtypes of individual cell types were annotated and shown.

To demonstrate the reliability and clinical efficacy of the bulk transcriptome-based CRscore, ten public datasets containing microarray data were obtained from the GEO database (**Additional file 1**: **Table S1**). The raw data were log2-transformed and normalized using the Robust Multiple Array Average (RMA) of the affy R package. Differential analysis was performed based on the “limma” R package.

### Calculation of the CRscore based on the differential circadian‐related genes

Circadian‐related genes (CRGs) were extracted according to previous publications (17). DEGs for each cell cluster were determined utilizing the FindAllMarkers function from the Seurat package with an adjusted p-value less than 0.05. Differentially expressed CRGs were finally determined after integration.

To assess the degree of circadian rhythm disruption in each cell or sample, we utilized a previously reported algorithm that calculates the change in signal-to-noise ratio for different genes and cells based on the expression profile of CRGs (18). Briefly, after calculating the average normalized values for microarray data and TPM for scRNAseq, a random sampling approach with 1000 repeats was utilized to divide all genes into fifty expression bins, and the random signature genes from each expression bin were then selected. According to the pertinent formulas, the CRscore belonging to normal distributions or mixtures of normal distributions were determined.

The threshold for the CRscore in single-cells was set at 75% based on quartiles, whereas the median value was chosen for microarray data.

### Cell–cell communication analysis

Cell–cell interaction analysis was performed using the CellphoneDB package (www.cellphonedb.org), which investigates the ligand-receptor interactions on the basis of the expression of ligand/receptor among different cell types (clusters). The group-separated scRNA-seq counts matrix identified by Seurat was utilized for further investigation. Clusters containing fewer than 50 cells were eliminated. The number of statistical iterations was set to 1000, and genes expressed by fewer than 10% of each cluster cell were eliminated. The CellPhoneDB repository informs subsequent interactions. *P-*value < 0.05 was deemed statistically significant for cell-cell interactions. the level of average receptor expression in a cluster and the level of average ligand expression in the interacting cluster were calculated. Dot plots were employed to illustrate the difference between the mean values of ligand-receptor interactions in low- and high-CRscore groups.

### SCENIC analysis for different CRscore groups

The python-based pySCENIC package (version: 0.12.0) was utilized to investigate the gene regulatory network of transcription factors (TFs) in AD with default parameters (19). First, the GENIE3 algorithm was performed to identify modules of co-expressed genes and transcription factors (TFs) from the counts matrix of scRNA-seq data. Then, these modules are trimmed by performing cis-regulatory motif identification on the probable target genes using RcisTarget, so that only genes containing the binding motif for the TF are retained in the module. This set of TFs and their potential target genes is known as a regulon. Finally, the activity of each regulon is quantified in each cell utilizing a recovery analysis in which all genes in a given cell are scored from low to high expression and plotted against the number of genes in a given regulon that can be recovered in that cell. Finally, the differences in TF activity scores between the low- and high-CRscore groups were visualized using a heatmap.

### Pseudotime trajectory analysis

Pseudotime trajectory analysis was conducted using the R package Monocle 3 (20). Seurat object was converted to a cd object using the new_cell_data_set function, followed by normalization using the preprocess_cds function. Then, UMAP dimensional reduction analysis and cell clustering were applied for this object with the reduce_dimension and cluster_cell functions from the Monocle 3 R package, respectively. With the use of the learn_graph function, a primary graph was trained from previously dimensional reduction and visualized to depict the development trajectory. The graph was then employed to sort cells through the developmental program (order_cells function) based on the cells expressing chosen markers as the trajectory root cells.

### Functional enrichment analysis

Differentially expressed CRGs were enrolled in the functional enrichment analysis. With the usage of the STRING database, functional network and gene connectivity data were collected. STRING provides gene connection data based on multiple pieces of evidence (direct interaction, co-localization, gene-regulation, and co-citation), grouping closely linked genes with the highest degree of certainty (0.9 interaction score). The output data from STRING database was then analyzed using the R package iGraph, Subsequently, a network analysis of the retrieved connectivity data was performed to highlight subnetworks or neighborhoods based on the random walk approach. The generated neighborhoods were enriched for the Kyoto Encyclopedia of Genes and Genomes (KEGG) using the clusterProfiler R package (21).

Activity scores of classical cancer-related signaling pathways between low- and high-CRscore groups were calculated using the R package of progeny, as previously reported (22).

Single-cell metabolic activity was quantified using the R package scMetabolism. We selected the VISION pipeline to analyze the signature scores of KEGG metabolic gene sets due to its quick execution and applicability to huge datasets. The resulting matrix of signature scores for metabolic gene sets was also incorporated into the Seurat object, and the differences in metabolic activity between low- and high-CRscore groups were visualized using a heatmap.

### Gene set variation analysis

On the basis of the “GSVA” R package, the Gene Set Variation Analysis (GSVA) enrichment was performed to examine the heterogeneity of a variety of biological processes. The hallmark gene set was extracted from the molecular signature database (MSigDB, http://www.gsea-msigdb.org/gsea/msigdb/index.jsp). The limma R package was utilized to identify the significantly different biological functions between the low‐ and high‐CRscore groups, and absolute t-values with a GSVA score more than 2 were deemed statistically significant.

### Estimation of the immune microenvironment in public bulk RNA−sequence datasets

The immune infiltrating levels in public bulk RNAsequence datasets were determined using the ssGSEA, MCPcounter, xCell, ABIS, and ESTIMATE algorithms, as previously described (23). Briefly, the proportions of various immune cell subtypes in each sample were evaluated utilizing global marker genes, and the aforementioned algorithms were conducted to calculate fractional enrichment or a relative percentage for each immune cell subset. The Wilcoxon rank-sum test was utilized to compare the degree of immunological infiltration between groups. Multiple algorithm-based immune infiltration levels were visualized using a heatmap. In addition, the ImmuneScore between groups were computed using the “ESTIMATE” R package to assess the immunological microenvironment of AD patients. Finally, the levels of expression of various immunoregulatory genes, such as MHC-I, MHC-II, immunoinhibitor, chemokine, and chemokine receptor, were determined in order to investigate the differences in immunological competence between low- and high-CRscore groups.

### Connectivity map analysis

A popular approach, CMap analysis, was conducted to predict treatments for individuals based on similar gene expression profiles (24). In this investigation, the top 100 up-regulated and 100 down-regulated genes in AD patients with high-risk and low-risk, respectively, were chosen as input data. The drug signature information acquired from the CMap database was selected as the preferred drug information. Then, the similarity of gene expression and drug signatures was compared using the eXtreme Sum (XSum) algorithm, and the CMap scores were computed to evaluate potential therapeutic drugs targeting AD patients at different risks.

### Generation of characteristic CRGs based on machine learning-dependent integrative approaches

As previously reported (25), gene expression profiles were transformed into z-scores across all datasets to improve comparability between various cohorts. T We combined 12 machine learning algorithms and generated 113 algorithm combinations to further filter consensus CRGs with good accuracy and stability. The integrative algorithms included random forest (RF), least absolute shrinkage and selection operator (Lasso), Ridge, elastic network (Enet), Stepglm, support vector machine (SVM), glmBoost, Linear Discriminant Analysis (LDA), Gradient Boosting Machine (GBM), eXtreme Gradient Boosting (XGBoost), and NaiveBayes. The process for creating signatures was as follows: (a) Expression profiles of CRGs with differential expression found in single-cell data were chosen. (b) Then, 113 algorithm combinations were performed on the differentially expressed CRGs to fit diagnostic models based on the 10‐fold cross‐validation in the GSE63060 dataset; (c) All models were verified in four validation datasets (GSE122063, GSE140829, GSE33000, and GSE36990); (d) For each model, the area under receiver operating characteristic curve (AUC) value was determined across all validation datasets, and the model with the highest mean AUC was deemed optimal; (e) In GSE5281, Characteristic CRGs generated from the optimal machine learning model were fitted into the Lasso model to obtain the corresponding coefficients for the most predictive genes; (f) In GSE5281, Characteristic CRGs generated from the optimal machine learning model were fitted into the Lasso model to obtain the corresponding coefficients of the most predictive genes; (g) The performance of riskScore was compared with other clinical features in GSE5281, GSE28146, GSE33000, GSE122063, GSE140829, and GSE36980.

### Primary culture of cortical neurons

Cultures of cortical neurons were performed in embryonic Sprague-Dawley rats (16-18 days) euthanized according to previous reported (26). Briefly, cortices from embryonic rats were extracted, chopped into 2 mm segments, and trypsinized for 20 min at 37°C using 0.25% trypsin solution (Gibco, NY, USA). After washing with PBS, the digested tissue was mechanically dissociated with a sterile fine bore glass pasteur pipette in the presence of a small amount of DNase1. The cell suspension was filtered through a 22-microm strainer to eliminate as many cell aggregations as possible. Neurons were seeded on 100 μg/ml poly-L-lysine coated 6-well plates at a density of 3 × 10^6^ cells/per well, and then incubated with a neurobasal medium (Gibco, NY, USA) containing 2% B27 supplement (Gibco, NY, USA), 0.5-mM L-glutamine (Gibco, NY, USA), and 50 U/ml of penicillin-streptomycin (Gibco, NY, USA). The entire culture medium was changed after 8 h, and half of the medium was renewed every 2-3 days. The cortical neurons cultured on days 7-9 at 37°C and 5% CO_2_ were utilized for further analysis. This work was authorized by the Institutional Animal Care and Use Committee of Fujian Medical University and conducted in accordance with the Guidelines for the Care and Use of Laboratory Animals.

### The preparation of Aβ1-42 oligomer and establishment of an *in vitro* model of AD

Aβ1-42 was initially dissolved in pre-cooled hexafluoroisopropanol (HFIP) to a concentration of 1mmol/L. After sonication, incubation at room temperature, and lyophilization, the obtained Aβ1-42 peptide membrane was re-dissolved in dimethyl sulfoxide (DMSO) (Gibco, NY, USA) and the F-12 medium (Gibco, NY, USA) was added to the A1-42 peptide membrane and then incubated overnight at 4°C to sustain oligomeric conditions. To simulate the *in vitro* model of AD, primary cortical neurons (DIV 7-8) grown in 6-well plates were treated with 20 umol/L Aβ1-42 oligomer for 12 hours at 37°C. Subsequently, the culture medium was replaced with standard neurobasal medium, and the cells were cultured in a 37°C incubator with 5% CO_2_.

### Real-time RT-PCR analysis

The TRIzol reagent (ThermoFisher Scientific, MA, USA) was utilized to extract total RNA from primary cortical neurons grown in 6-well plates for 7-8 days according to the manufacturer’s instructions. Then, the RevertAid First Strand cDNA Synthesis Kit (Thermo Fisher Scientific, MA, USA) was used to reverse the total RNA into complementary DNA (cDNA). The primers employed for RT-PCR analysis were as follows: GLRX: forward, 5′- TGTGAACTGCAAGATTCAGTCTG′, reverse, 5′-TGTTGTAAATAATCTTGAATCGCAT-3’; MEF2C: forward, 5′-AGATATTGATCTAATGATCAGCAGG-3′, reverse, 5′- TGTCACACCAGGAGACATACTATTC-3’; PSMA5: forward, 5′-ATGTCTAGCTGTGGAGAAGAGAATT-3′, reverse, 5′- TTGTCTCATTATAGGTGAACCAGTG-3′; NR4A1: forward, 5′-GCTTCTTCAAGCGCACAGTAC-3′, reverse, 5′-GAATGAGGGACGTGAGGAGATT-3′; SEC61G: forward, 5′-TGGATCAGGTAATGCAGTTTGT-3′, reverse, 5′- GTTATTAATAGGGATGTGGATCAGT-3′; RGS1: forward, 5′-TGGAATGGACATGAAAGCATATC-3′, reverse, 5′-TGTTCTCTTCACTGAATTCAGACTT-3′; CEBPB: forward, 5′-CACGCTGCGGAACTTGTT-3′, reverse, 5′-TGATCCGGATTGCATCAAGT-3′. The qRT-PCR was conducted using a SYBR^®^ Premix Ex Taq™ II (Takara, Shiga, Japan) in an ABI 7500 Real-Time PCR system (Applied Biosystems, CA, USA). The quantity of mRNA was estimated using cycle threshold (CT) values that were normalized against the amount of rat β-actin mRNA. ΔCT was calculated by subtracting the CT value of the β-actin from the CT value of respective target gene. Further calculation was conducted using the2^-ΔΔCT^ method, and the results were characterized as a relative increase in mRNA expression compared to control values.

### Other statistical analysis

All statistical analyses and visualizations were conducted utilizing the R 4.1.0 software.

Continuous variables were visualized as means ± standard deviations (SD), and Counts or percentages (%) were employed to summarize categorical variables. The chi‐square test was utilized to compare the frequencies to categorical variables. The Wilcoxon sum-rank test or t-test was applied for comparing the difference of continuous variables between two groups. The ROC curve analysis was performed to evaluating the performance of binary categorical variables. A two-sided p-value less than 0.05 was considered to be statistically significant.

## Results

### CRD-based single-cell transcription atlas in AD immune microenvironment

**Figure 1:**
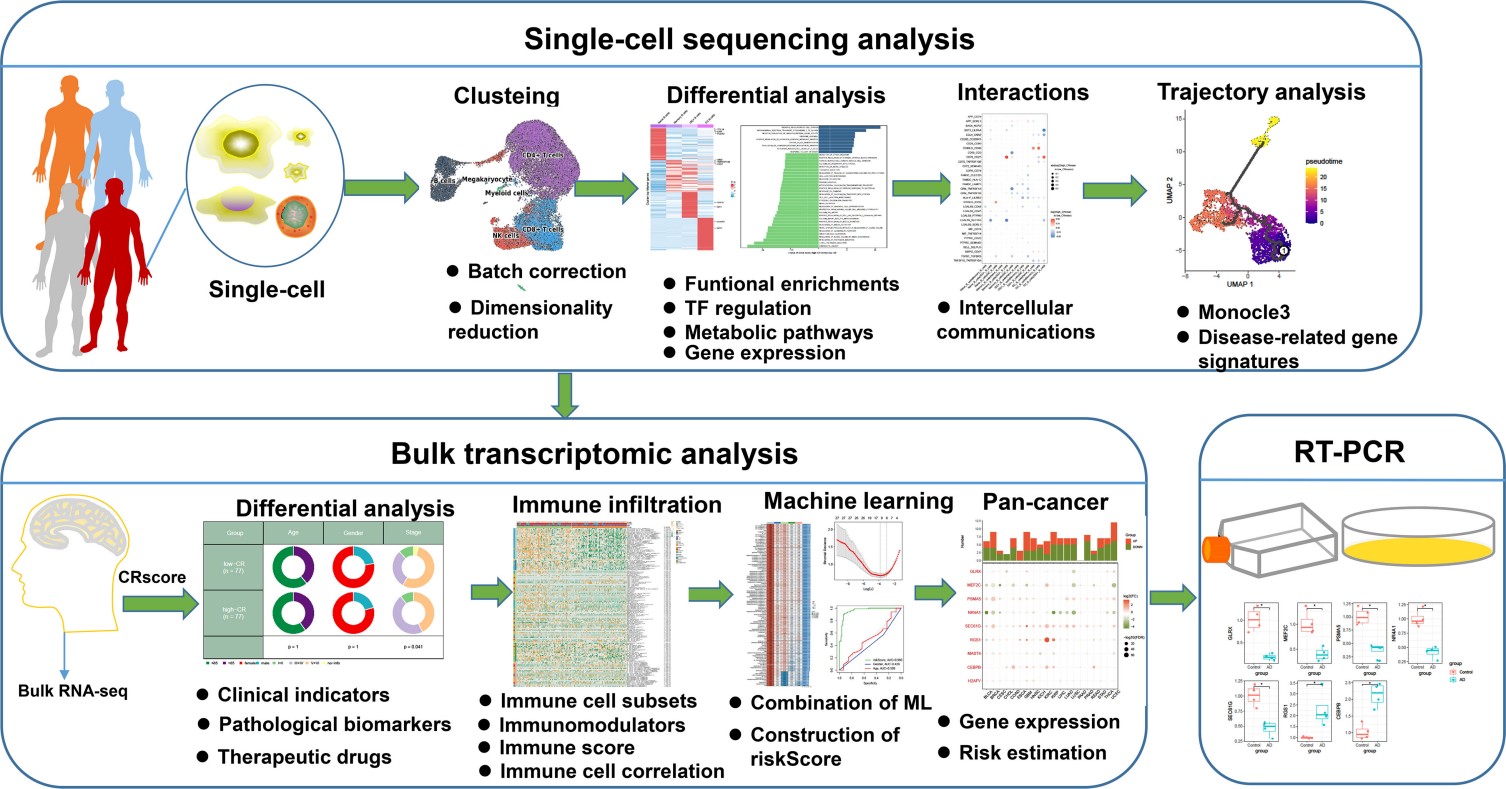
The flowchart of this study. *p < 0.05.

**Figure 2:**
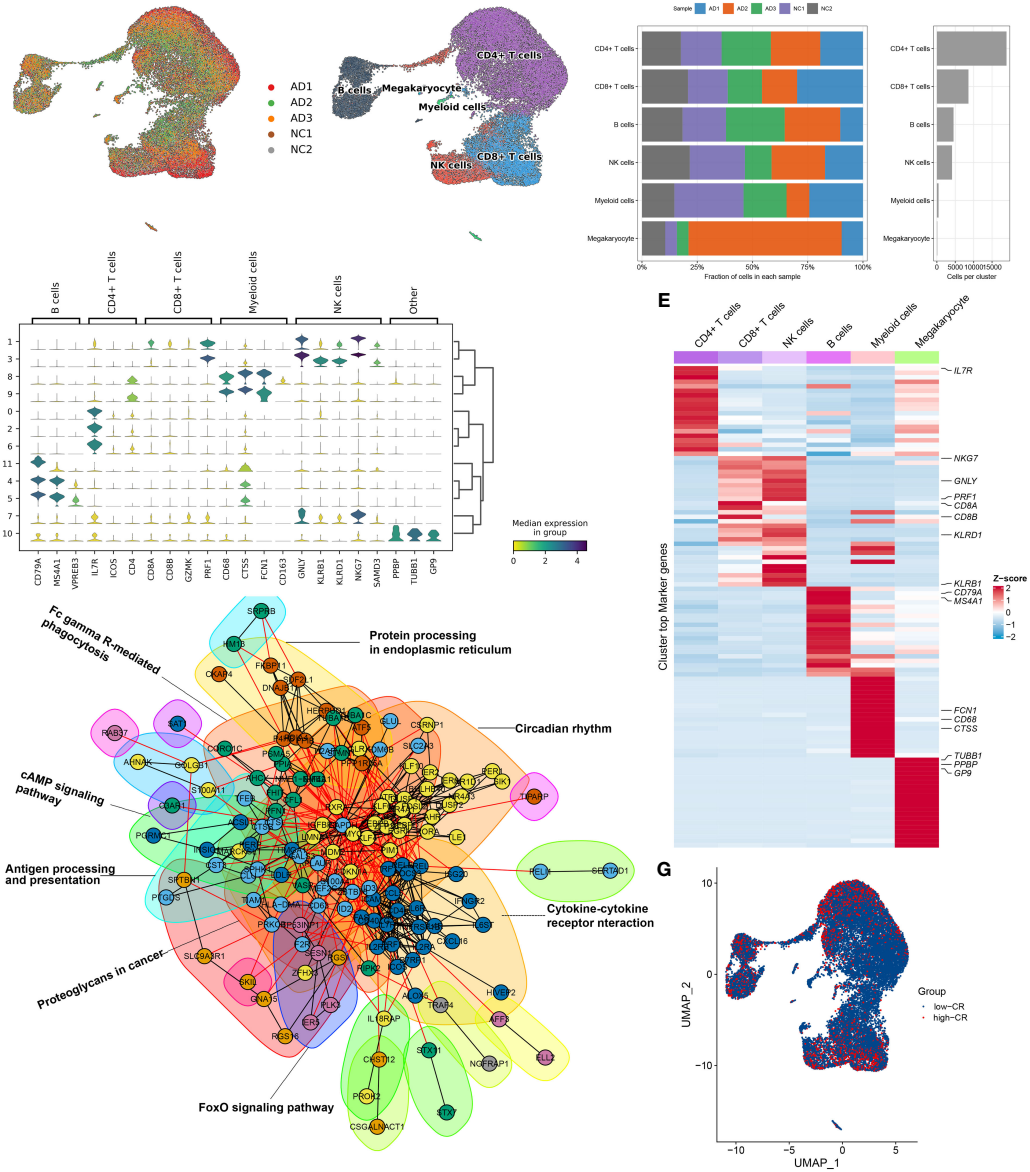
Overview of CRD heterogeneity in AD peripheral blood samples. **(A, B)** Sample distribution **(A)** and cell type annotations **(B)** by using the uniform manifold approximation and projection (UMAP) plot of 36725 cells. **(C)** Violin plot of marker genes for distinct cell types. Color scale represents the median expression of marker genes in each cell type, and the violin size indicates the fraction of cells expressing the marker genes inside each cell cluster. **(D)** Bar plots exhibit the proportion of 6 main cell types across different samples (left panel), as well as the total cell number in each cell type (right panel). **(E)** The heat map exhibits the expression profiles of the top 20 feature genes in each cell type. Red color represents up-regulation, and blue color represents down-regulation. Highly expressed marker genes of each cell type are annotated. **(F)** Gene interaction networks of the differentially expressed CRGs from ScRNA-seq analysis are constructed using the STRING database. Subnetworks (Neighborhoods) are colored and labeled with enriched functional categories. Gray and red lines represent connections within a neighborhood and between neighborhoods, respectively. **(G)** A global landscape of CRscore in AD samples was displayed using the UMAP plot.

To clarify the processes underlying CRD during AD, we initially re-analyzed a previously published scRNA-seq dataset (GSE181279) that included 2 normal and 3 AD peripheral blood samples using the Seurat approach. The flowchart of this study was shown in **Figure 1**. After quality control filtering, a total of 18400 unique genes were obtained from 36725 cells originating from five samples (**Figure 2A**). After normalizing gene expression, we performed the PCA and clustered cells using UMAP‐based clustering on the informative PCA space (n = 15), and a total of 12 distinct cell clusters were determined on the basis of highly variable genes (**Figure S1A**). These cell clusters were classified into recognized cell types based on multiple cell markers: CD4^+^ T cells (18929 cells), CD8^+^ T cells (8577 cells), NK cells (4113 cells), B cells (4535 cells), myeloid cells (457 cells), and megakaryocyte (114 cells) (**Figures 2B, C**). Among them, approximately 14013 cells belonged to control samples, while 22712 cells originated from AD patients (**Figure S1B**). The proportions of distinct cell types in each sample are depicted in **Figure 2D**, revealing that CD4^+^ T cells, CD8^+^ T cells, B cells, and megakaryocyte were abundant in AD patients. **Figure 2E** exhibits the expression landscapes of the top 20 feature genes in each cell subtype, indicating that these unique markers could differentiate cell subtypes precisely.

Next, we crossed 2091 CRGs with 1098 AD-related DEGs and finally determined 201 differentially expressed CRGs. On the basis of the STRING database, we performed interaction analysis to predict the relationships among these 201 CRGs and identified corresponding subnetworks or neighborhoods based on functional annotation analysis. Pathway enrichment analysis revealed that these differentially expressed CRGs primarily affect the cytokine-cytokine receptor interaction, cAMP signaling pathway, FoxO signaling pathway, circadian rhythm, antigen processing and presentation, and protein processing in endoplasmic reticulum (**Figure 2F**). It was reported that circadian rhythm disruption is closely related to the pathogenesis of AD (6, 27, 28). While the underlying mechanisms remain largely unknown. Therefore, we subsequently focused on the relationship between cells of AD sample origin and CRD. A scoring algorithm was employed to quantify the CRscore in AD cells. As shown in **Figure 2G** and **Figure S1C**, CRscore was significantly greater in the various clusters (clusters 1, 2, 3, 4, and 5). Obviously, B cells exhibited a higher degree of CRscore, that is, a rather stronger CR, compared with the other cell subtypes. In addition, the AD cells were classified into high- and low CR groups in terms of CRscore (**Figure S1D**). Meanwhile, 9 cyclin-related genes (CCNC, CCND2, CCND3, CCNH, CCNI, CCNK, CCNL1, CCNL2, and CCNT1) were significantly upregulated in low-CR AD cells (**Figure S1E**), suggesting cell cycle activation was significant in low-CR group.

### CRscore-based B cell subsets revealed by scRNA-seq in AD

**Figure 3:**
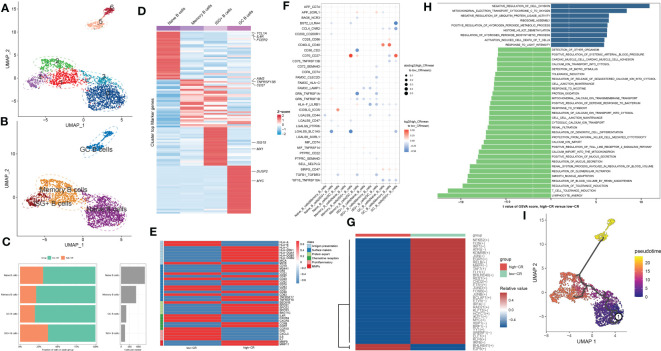
CRD altered the features of B cells. **(A, B)** UMAP visualization of 2811 B cells from AD samples, colored by cell clusters **(A)** and cell subtypes **(B)**. **(C)** The proportion of 4 B cell subtypes in the low- and high-CR groups and the total cell number in each B cell subtype (right panel) are exhibited using bar plots. **(D)** Heatmap exhibits the expression profiles of the top 30 feature genes in each B cell subtype. Red color and blue color represent up-regulated and down-regulated genes, respectively. The highly expressed marker genes of each B cell subtype are annotated. **(E)** Heatmap displaying the expression profiles of genes associated with antigen presentation, surface markers, protein export, chemokine receptors, pro-inflammatory and MMPs in B cells with low- and high-CRscore. **(F)** Dot plot shows the intercellular interactions between low- and high-CR B cells in AD patients. **(G)** Heatmap indicates the significantly expressed TFs between low- and high-CR B cells in AD patients. **(H)** GSVA exhibits the abundant biological functions between low- and high-CR B cells in AD patients. The higher the t value, the more significant the biological functions. **(I)** Trajectory analysis reveals the differentiation process of B cells.

To generate a comprehensive transcriptional atlas of B cells in AD, we extracted 2811 B cells originating from AD samples for further sub-clustering analysis, and a total of 7 distinct cell clusters were finally identified (**Figure 3A**). These cell clusters were further subdivided into four B-cell subgroups, including naive B cells (1384 cells), memory B cells (873 cells), interferon-stimulated genes (ISG^+^) B cells (230 cells), and germinal center (GC) B cells (300 cells) (**Figure 3B**). The average number and cell proportion of B cell subsets showed a considerable difference between low- and high-CR groups (**Figure 3C**). **Figure 3D** displays the expression landscapes of the top 30 signature genes and marker genes in each cell type, indicating a significantly distinct expression among these four B-cell subgroups. It was observed that the expression of antigen presenting and surface markers, apart from protein export and MMPs, was significantly greater in the high-CR group relative to the low-CR group, while the low-CR group exhibited a notable higher expression of proinflammatory and chemokine receptors (**Figure 3E**). CellPhoneDB-based cell-cell commination analysis revealed that GC B cells in the high-CR group frequently interacted with naive B cells, memory B cells, and ISG^+^ B cells through several ligand-receptor pairs such as CD40LG_CD40 and CD70_CD27. Meanwhile, memory B cells in the high-CR group could also interact with GC B cells through HLA-F_LILRB1, CD70_CD27, and APP_SORL1 (**Figure 3F**). Furthermore, the SCENIC analysis was conducted to determine the alterations in TFs from low-CR to high-CR. It was found that the high-CR group exhibited activated TFs (including NFKB2, FOS, IRF1, ATF5, KDM5B, JUN, EGR1, RELB, MAFF, TAF7, FLI1, BHLHE40, REST, CHD2, ETS1, JUND, FOSB, JUNB, BCLAF1, ETV5, IRF4, RAD21, KLF12, HCFC1, IKZF1, MYC, XBP1, BRF1, YY1, SREBF1, ELF1, KLF6, and IRF8) and inhibited TFs (including BHLHE41 and E2F6) (**Figure 3G**), suggesting that CRD is closely associated with TF dysregulation in AD patients.

To estimate the differences in biological functions between AD patients with different levels of CR, we performed GSVA and detected that the metabolic process, mitochondrial electron transport, ribosome assembly, ubiquitin protein ligase activity, and negative regulation of cell division were the enriched signatures in high-CR group (**Figure 3H**). Further comparison using metabolic pathway enrichment analysis demonstrated that the metabolic processes associated with ascorbate and aldarate, glycosphingolipid biosynthesis, other glycan degradation, tryptophan, β-alanine, glycerophospholipid, pentose phosphate, and glutamate were prominently upregulated in the high-CR group (**Figure S2A**). The pseudotime analysis depicted the temporal sequence of distinct B cells differentiation. Naive B cells were mainly localized at the start of the differentiation pathway, while GC B cells were prevalent at the end (**Figure 3I**). These findings revealed that naive B cells could convert into GC B cells. During this process, surface markers, chemokine receptors, and protein export related genes, were observed to be considerably altered (**Figure S2B**).

### CRscore-based CD4^+^ T cells exhibit distinct molecular characteristics in AD

**Figure 4:**
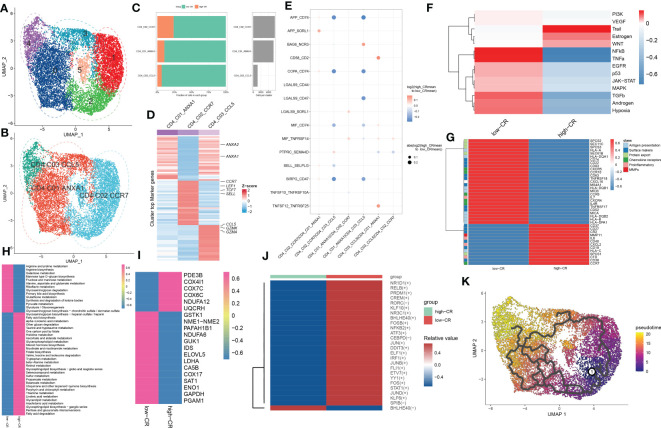
CRD-mediated CD4^+^ T cell subtypes impact AD molecular functions. **(A, B)** UMAP visualization of CD4^+^ T cells from AD samples, colored by cell clusters **(A)** and cell subtypes **(B)**. **(C)** The proportion of each defined CD4^+^ T cell subtype across low- and high-CR groups (left panel) and the total cell number in each CD4^+^ T cell subtype (right panel) are exhibited using bar plots. **(D)** Heatmap exhibits the expression profiles of the top 30 feature genes in each CD4^+^ T cell subtype. Red color and blue color represent up-regulated and down-regulated genes, respectively. **(E)** Dot plot shows differential cell-cell interactions between low- and high-CR CD4^+^ T cells in AD samples. **(F)** Heatmap displays differences in the activity of 14 classic pathological pathways between low- and high-CR CD4^+^ T cells in AD samples. **(G)** Heatmap displays the expression profiles of genes associated with antigen presentation, surface markers, protein export, chemokine receptors, pro-inflammatory and MMPs in CD4^+^ T cells with low- and high-CRscore. **(H–J)** Heatmap exhibits significantly different metabolic pathways **(H)** and genes **(I)**, as well as TFs **(J)**. **(K)** Trajectory analysis reveals the differentiation process of CD4^+^ T cells.

As we were aware, AD patients exhibited higher proportions of CD4^+^ T cells. In the current study, the presence of CRscore-based CD4^+^ T cells in AD patients encouraged us to explore their subtype status. Six distinct subgroups in the AD CD4^+^ T cell cluster were determined on basis of UMAP analysis (**Figure 4A**). Previously known markers, including ANXA1, ANXA2, CCR7, LEF1, TCF7, SELL, CCL5, GZMK, and GZMA could accurately subdivide CD4^+^ T cells into three subgroups, including CD4_C01_ANXA1 (5318 cells), CD4_C02_CCR7 (5645 cells), and CD4_C03_CCL5 (1148 cells) (**Figure 4B**). The corresponding abundances for each cluster between low- and high-CR groups were discrepant (**Figure 4C**). Here, subpopulation marker genes and the top thirty differentially expressed genes for each cluster are depicted in **Figure 4D**. For CD4+T cells, CD4_C01_ANXA1 was assigned to central memory CD^4+^ T cells, which were characterized by the expression of ANXA1 and ANXA2; CD4_C02_CCR7 with high expression of naive markers (CCR7, SELL, TCF7, and LEF1) represented naive CD4+T cells. Meanwhile, CD4_C03_CCL5 exhibited increased expression of cytotoxic effectors (GZMK, GZMA, and CCL5) revealing that they were closely related to cytotoxic effector CD4+ T cells.

We next compared the cell-cell interactions of CD4^+^ T cell subgroups between groups using CellPhoneDB. It was found that multiple ligand-receptor pairs, including APP_CD74, COPA_CD74, MIF_CD74, and SIRPG_CD47, were active in the low-CR group from CD4_C01_ANXA1 and CD4_C02_CCR7 cells to CD4_C03_CCL5 cells (**Figure 4E**). In addition, the PROGENy analysis revealed that the low-CR group showed significant upregulation in 11 out of 14 classic disease progression-related pathways (**Figure 4F**), suggesting that low levels of CRscore in CD4^+^ T cells seem to be more closely related to AD progression. Notably, the increased expression of most genes associated with antigen presentation (HLA-A, HLA-B, HLA-DPA1, HLA-DQA1, HLA-DQB1, HLA-DQB2, MICA, and MICB), surface markers (MS4A1, CD22, CD52, CD74, CD83, CD63, TNFRSF17, TNFRSF18, and SEC61B), chemokine receptors (IL4R, CXCR4, CXCR5, CCR6, and CCR10), and protein export (SEC61B, SPCS2, SPCS3, and SEC11C) were observed in low-CR group (**Figure 4G**).

Meanwhile, GSVA revealed that the low-CR group was predominantly involved in weakening circadian rhythm, cellular homeostasis, macrophage migration, RNA migration, and toll like receptor2 signaling pathway (**Figure S3A**). Subsequently, we further comprehensively assessed the differences in metabolic pathways between CD4^+^ T cells with different levels of CRscore. We observed that 44 out of 85 metabolic pathways were notably different between low- and high-CR groups. Interestingly, CD4^+^ T cells with a low CRscore enriched in a larger number of metabolic pathways and genes (**Figures 4H, I**). We further performed the SCENIC analysis to estimate the upstream TFs for CRD during the differentiation of CD4^+^ T cells. The results revealed that the responsible TFs in the low-CR group were CR1D1, RELB, PRDM1, CREM, RORC, KLF10, NR3C1, BHLHE40, FOSB, NFKB2, ATF3, CEBPD, JUN, DDIT3, ELF1, IRF1, JUNB, FLI1, ETV7, YY1, FOS, STAT1, JUND, KLF6, and SPIB. As the modulators of cAMP responsive element, JUNB and CREM have been reported to suppress the functions of CD4^+^ regulatory T cells (29, 30) (**Figure 4J**). To confirm that the population of CD4^+^ T cells consisted of groupings of cells positioned in distinct stages along the course of differentiation, we cross-validated our findings using the Monocle 3 approach. As a result, we found that the differentiation state began to alter around the CD4_C02_CCR7 cluster, while the CD4_C03_CCL5 was located at the terminal of developmental stage (**Figure 4K**). On the basis of the starting stage of the differentiation, several surface markers (CR2, CD63, CD40, CD37, CD27, and CD38) and CCR7 were decreased, whereas other surface markers (MS4A1, CD63, and TNFRSF18), chemokine receptors (CXCR4, CXCR5, and CCR6), and protein export genes (SEC61B, SPCS1, SPCS2, and SPCS3) were increased along the course of differentiation (**Figure S3B**).

### CRscore-based CD8^+^ T cells display dysregulation in signaling, intercellular communications, and differentiation during AD

**Figure 5:**
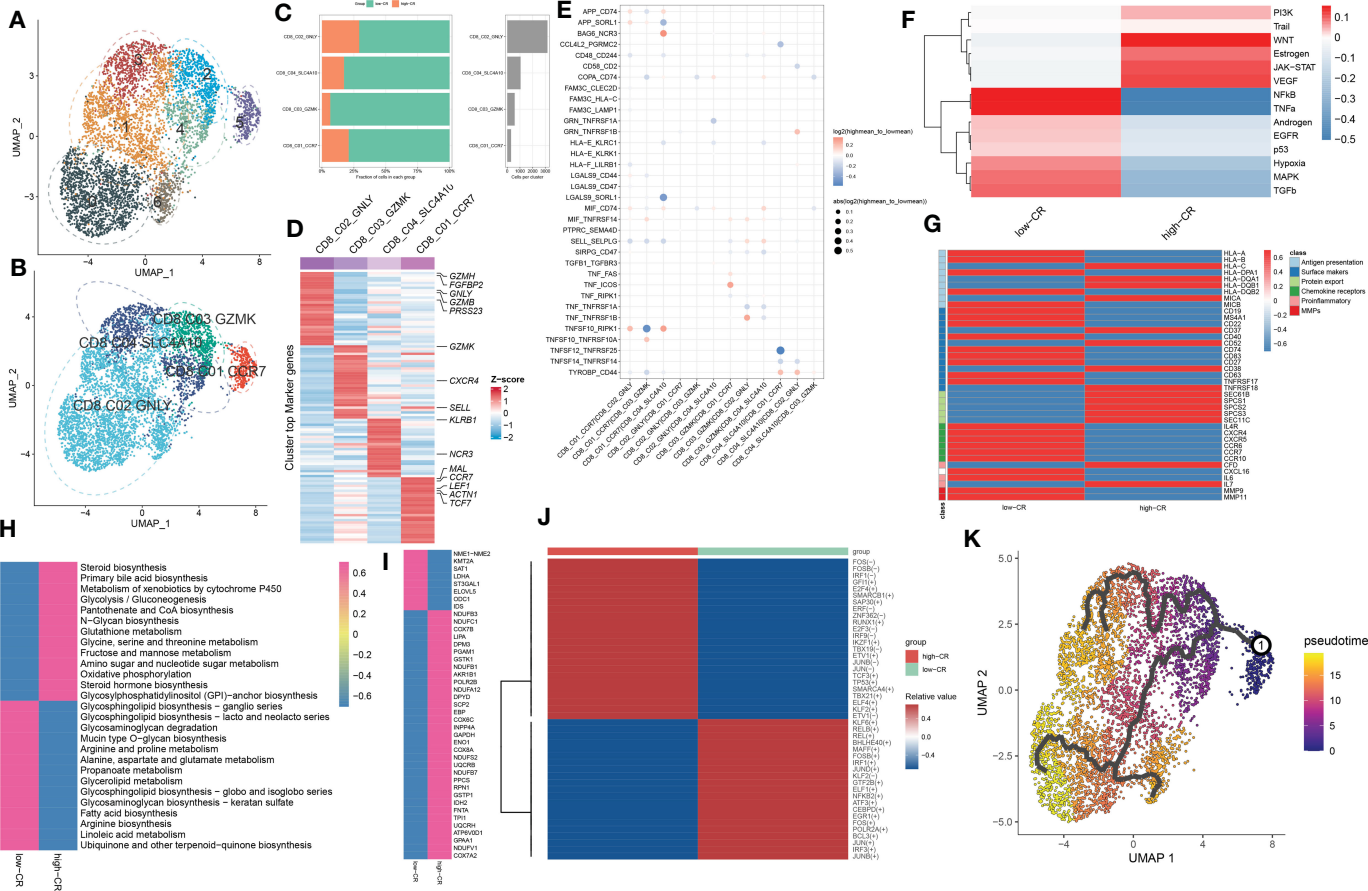
CRscore-based CD8^+^ T cells. **(A, B)** UMAP visualization of CD8^+^ T cells from AD samples, Distinct cell clusters **(A)** and cell types **(B)** are labeled with different colors. **(C)** Represent bar plots depict the proportion of each defined CD8^+^ T cell subtype across low- and high-CR groups (left panel) and the total cell number in each CD8^+^ T cell subtype (right panel). **(D)** Represent heatmap depicts the expression landscapes of top 30 feature genes in each CD8^+^ T cell subtype. Red color and blue color represent up-regulated and down-regulated genes, respectively. **(E)** Represent dot plot depicts differential intercellular communications between low- and high-CR CD4^+^ T cells in AD samples. **(F)** Represent heatmap displays difference in the activity of 14 classic pathological pathways between low- and high-CR CD8^+^ T cells in AD samples. **(G)** Represent heatmap exhibits the expression landscapes of genes associated with antigen presentation, surface markers, protein export, chemokine receptors, proinflammatory and MMPs in CD8^+^ T cells with low- and high-CRscore. **(H–J)** Represent heatmap displays notably different metabolic pathways **(H)** and genes **(I)**, as well as TFs **(J)**. **(K)** Trajectory analysis reveals the differentiation process of CD4^+^ T cells.

For elucidating the heterogeneity among CD8^+^ T cells, 5250 CD8+ T cells were re-clustered into 7 clusters using UMAP analysis (**Figure 5A**), which were further grouped into CD8_C01_CCR7 (naive cells, 301 cells), CD8_C02_GNLY (TEMRA cells, 3168 cells), CD8_C03_GZMK (cytotoxic effector cells, 585 cells), and CD8_C04_SLC4A10 (MAIT cells, 1050 cells) (**Figure 5B**). Compared to samples from the high-CR group, the number and proportions of these four subtypes decreased in the low-CR group (**Figure 5C**). **Figure 5D** depicts the top 30 differentially expressed genes for each cluster. Cell-cell interactions analysis of CD8^+^ T cells subsets between low-CR and high-CR groups suggested that the low-CR group displayed the increased activity of APP_CD74, APP_SORL1, COPA_CD74, HLA-E_KLRC1, LGALS9_SORL1, SELL_SELPLG, TNFSF10_RIPK1, and TYROBP_CD44 ligand-receptor pairs from CD8_C01_CCR7 cells to the rest of CD8+ T cell subtypes. In addition, the enrichment of CCL4L2_PGRMC2, CD58_CD2, TNFSF12_TNFRSF25, and TNFSF14_TNFRSF14 in low-CR group proved the potential role of CD8_C04_SLC4A10 in recruiting the other three subsets of CD8+ T cells (**Figure 5E**). Moreover, the PROGENy analysis demonstrated that the classical pathogenic pathways, including NFkB, TNFα, androgen, EGFR, p53, hypoxia, MAPK, and TGFβ were significantly enriched in the low-CR group, whereas high-CR group was close associated with the activation of PI3K, WNT, estrogen, JAK-STAT, and VEGF signaling pathways (**Figure 5F**). Furthermore, the expression of most marker genes associated with antigen presentation, surface markers, chemokine receptors, proinflammatory, and MMPS, was reinforced in the low-CR group, except for protein export genes (**Figure 5G**).

To better elucidate the differences in molecular characteristics between these two groups, we performed GSVA and found the high-CR group exhibited the increment of immune response, immune cell differentiation, mitochondrial electron transport, and neuro-inflammatory response, whereas the low-CR group was mainly regulated by neuron apoptotic process, the activation of neutrophil, mast cells, and myeloid cells, and the lipoxygenase pathway (**Figure S4A**). Enrichment analysis of 85 metabolic pathways further revealed that 27 of them were substantially distinct between the low- and high-CR groups, with 13 metabolic pathways being activated in the high-CR group and the other 14 metabolic pathways being repressed in the low-CR group (**Figure 5H**). Consistently, the low-CR group also concentrated on more metabolic genes (**Figure 5I**). The SCENIC analysis was conducted to detect the differences in TFs between groups. It was found that 24 of the 45 dysregulated TFs were enriched in the low-CR group, including FOS, FOSB, IFR1, GFI1, E2F4, SMARCB1, SAP30, ERF, ZNF362, RUNX1, E2F3, IRF9, IKZF1, TBX19, ETV1, JUNB, JUN, TCF3, TP53, SMARCA4, TBX21, ELF4, KLF2, and ETV1 (**Figure 5J**). Subsequently, in the diffusion map pseudotime, CD8_C01_CCR7 cells were mainly located in the most undifferentiated status, the CD8_C02_GNLY, and CD8_C03_GZMK cells were located in a distinct downstream trajectory of the CD8_C01_CCR7 cluster, and the CD8_C04_SLC4A10 was located in the final differentiation stage (**Figure 5K**). Significant differences in gene expression distribution patterns were observed during the differentiation of CD8^+^ T cell subpopulations. For example, the surface marker CD27 and chemokine receptors (CXCR5 and CCR6, and CCR7) were prominently expressed in the undifferentiated status during the pseudotime process, while other surface markers (CD52, CD40, CD37, CD38, CD63, and TNFRSF18) and protein export markers (SEC61B, SPCS1, SPCS2, and SPCS3) were curtailed along the stage of CD8^+^ T cell differentiation (**Figure S4B**).

### Verification of the effectiveness and robustness of CRscore based on bulk transcriptomic datasets

**Figure 6:**
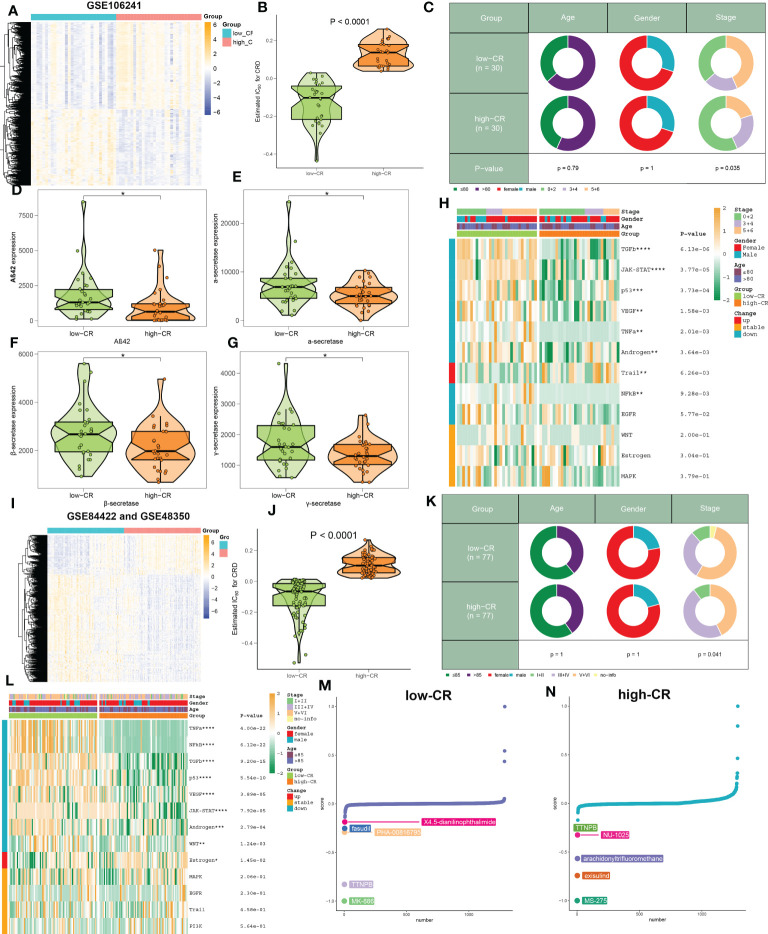
The clinical characteristics and predictive drugs of CRD groups in bulk transcriptomic datasets. **(A)** Representative heatmap depicts the expression landscapes of differentially expressed genes between AD patients with low- and high-CR in GSE106241. **(B)** The CRscore of low- and high-CR patients are compared in GSE106241. **(C)** Pie charts depict the differences in clinical characteristics (age, gender, and stage) between AD patients with low- and high-CR in GSE106241. **(D–G)** Representative violin plots depict the differences in AD pathological hallmarks (Aβ42, α-secretase, β-secretase, and γ-secretase) between AD patients with low- and high-CR in GSE106241. **(H)** Representative heatmap depicts the differences in 14 classic pathological pathways between AD patients with low- and high-CR in GSE106241. **(I)** Representative heatmap depicts the expression landscapes of differentially expressed genes between AD patients with low- and high-CR in the combined datasets of GSE84422 and GSE48350. **(J)** The CRscore of low- and high-CR patients are compared in GSE84422 and GSE48350. **(K)** Pie charts depict the differences in clinical characteristics (age, gender, and stage) between AD patients with low- and high-CR in GSE84422 and GSE48350. **(L)** Representative heatmap depicts the differences in 14 classic pathological pathways between AD patients with low- and high-CR in GSE84422 and GSE48350. **(M, N)** CMap study depicts the top five predictive drugs for patients with low- **(M)** and high-CR **(N)** in GSE84422 and GSE48350. *p < 0.05, **p < 0.01, ***p < 0.001, ****p < 0.0001.

Due to the insufficient samples involving the scRNA-seq dataset, we therefore collected several independent gene expression datasets related to AD to verify the performance of CRscore. In the GSE106241 dataset, we classified a total of 60 AD samples into low- and high-CR groups based on their median value of CRscore. The heatmap displayed the expression patterns of 1387 up-regulated and 1217 down-regulated DEGs between the low- and high-CR groups (**Figure 6A**). In these cases, samples with a high-CR had a significantly higher CRscore than those from the low-CR group (**Figure 6B**). More AD patients in advanced stages (*p* < 0.05) were observed in the low-CR group, while the distribution of age and gender was not statistically different (**Figure 6C**). In addition, relative to the high-CR group, the CRscore of the low-CR group exhibited increased activity of several typical AD-related pathological markers, including Aβ42, α-secretase, β-secretase, and γ-secretase (**Figures 6D–G**). Furthermore, among the 12 classical pathogenic pathways, the low-CR group was mainly responsible for the activation of TGFβ, JAK-STAT, p53, VEGF, TNFα, androgen, NFkB, and EGFR signaling pathways (**Figure 6H**). In the combined datasets of GSE84422 and GSE48350, 154 AD brain samples were divided into 77 low-CR and 77 high-CR groups in terms of CRscore, which exhibited notably distinct gene expression patterns and CRscore distribution between the two groups (**Figures 6I, J**). Consistently, the proportion of patients who belonged to stage V+VI was also higher in the low-CR group than that in the high-CR group (*p* < 0.05) (**Figure 6K**). Additionally, the activation of pathogenic pathways similar to those in the GSE106241 dataset can also be observed in the low-risk group of the combined dataset (**Figure 6L**). These findings indicated that the CRscore algorithm, which is based on the differentially expressed circadian gene feature, can accurately define the CR activity of AD patients in bulk transcriptome datasets.

Using the CMap database, we investigated prospective therapeutic drugs for low- and high-CR patients in order to evaluate individualized clinical treatments for AD patients. X4-5-dianilinophthalimide, fasudil, PHA-00816795, TTNPB, and MK-886 were the top five medicines with personalized therapeutic potential for the low-CR group (**Figure 6M**). While the RRNPB, NU-1025, arachidonyltrifluoromethane, exisulind, and MS-275 were the five most effective treatment medications for AD patients with a high CRscore (**Figure 6N**). Specifically, MK-866 and MS-275 had the lowest CMap scores in the low- and high-CR groups, respectively, showing the greatest treatment benefit in AD patients with varying CRscore.

### CRD involves the alterations in the immune microenvironment of AD

**Figure 7:**
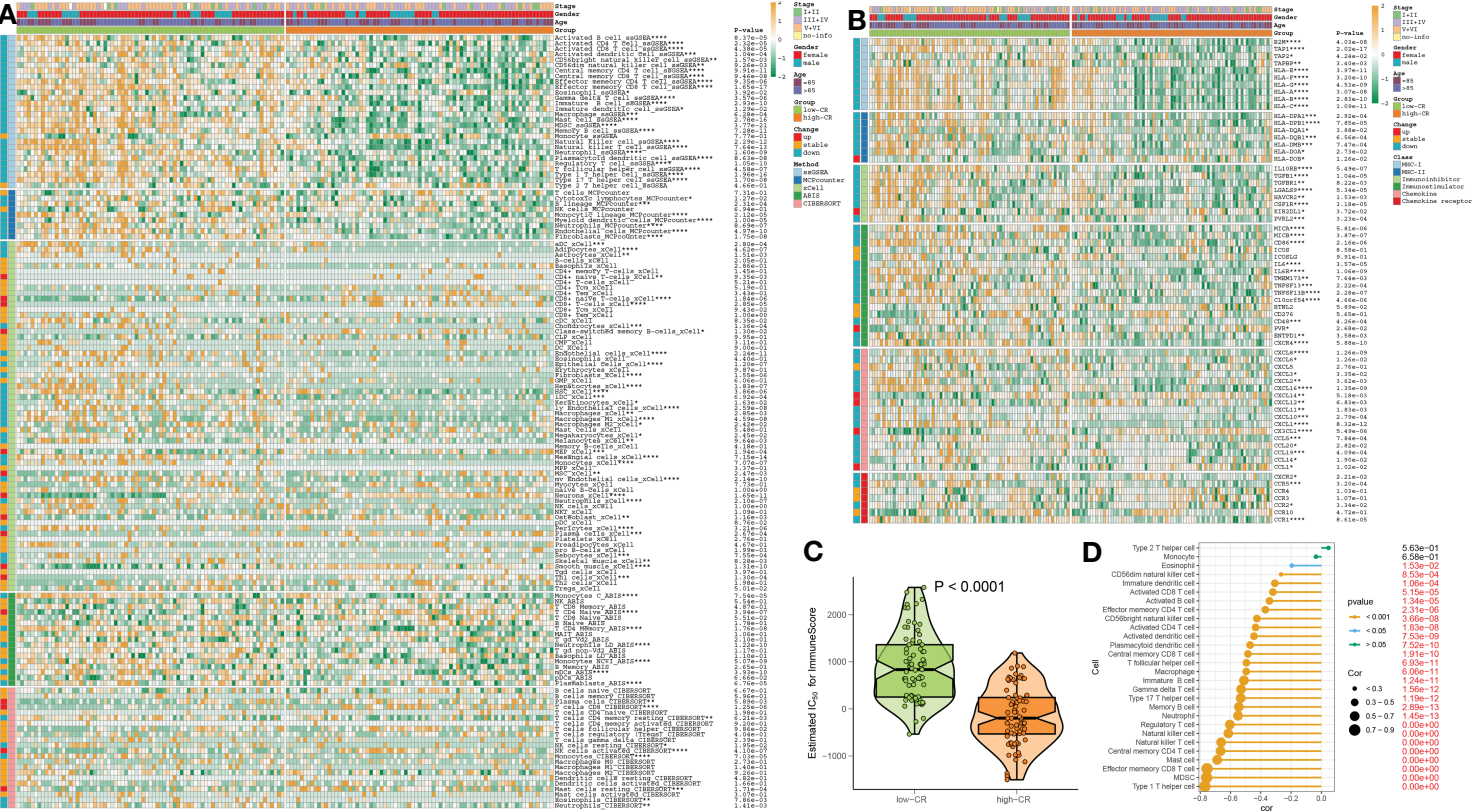
The immunological characteristics of AD patients with low- and high-CR in the combined datasets. **(A)** Heatmap exhibits the expression landscapes of infiltrated immune cells between AD patients with low- and high-CR based on the ssGSEA, MCPcounter, xCell, ABIS, and ESTIMATE algorithms. *p < 0.05, **p < 0.01, ***p < 0.001, ****p < 0.0001. **(B)** Heatmap exhibits the expression landscapes of immunoregulatory genes between AD patients with low- and high-CR. *p < 0.05, **p < 0.01, ***p < 0.001, ****p < 0.0001. **(C)** The immunological score of low- and high-CR patients are compared. **(D)** Lollipop plots depict the correlation between the CRscore and 28 immune cell subpopulations.

We then evaluated the association between CRD level and immune cell infiltration using the ssGSEA, MCPcounter, xCell, ABIS, and ESTIMATE algorithms in AD samples of the combined dataset, and found that the infiltration levels of immune cell subsets were significantly greater in patients with low-CR than those with high CRscore, as evidenced by the fact that the majority of immune cells based on the ssGSEA, MCPcounter, xCell, and ABIS algorithms were notably enriched in the low-scoring group (**Figure 7A**). Meanwhile, several immune chemokines, including CCL5 and CCL20, were elevated in the low-CR condition and were verified to perform critical roles in the accumulation of regulatory T cells. In addition, a generally elevated MHC-I, MHC-II, immunoinhibitor, immunostimulator, chemokine, and chemokine receptor was observed in low-CR patients, whereas the expression levels of HLA-DOB, KIR2DL1, PVR, CXCL14, CXCL12, CX3CL1, and CCL1 were higher in high-CR samples (**Figure 7B**). On the other hand, the level of immune score was pronouncedly enriched in patients with a low CRscore (**Figure 7C**). As demonstrated by the ssGSEA algorithm, a negative association occurred between the CRscore and the abundance of a majority of infiltrated immune cells, including NK cells, dendritic cells, CD8^+^ T cells, CD8^+^ T cells, B cells, macrophages, neutrophils, Th17, Treg, and Th1 cells (**Figure 7D**). The other dataset, GSE106241 was employed to validate the association between the CRscore and immunological characteristics in patients with AD. Similarly, low-CR patients also had higher levels of immune cell infiltration and immune response. These results revealed that CRD can induce a remodel of the immune microenvironment in AD patients, providing additional support for the observations made at the single‐cell level (**Figures S5A–D**).

### Integrative construction of a characteristic CRD signature

**Figure 8:**
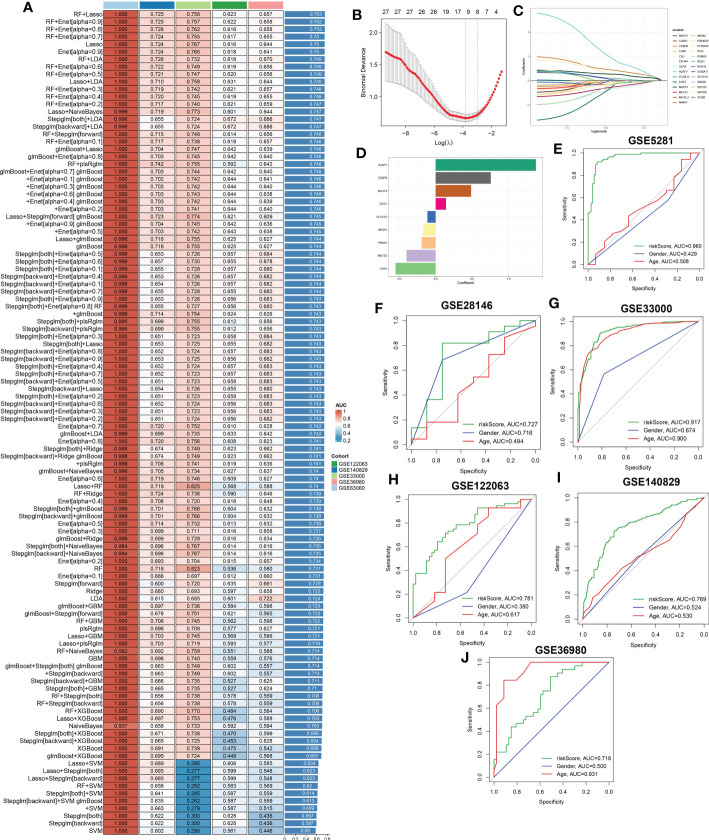
A characteristic CRD signature was constructed and validated based on the machine learning-based integrative model. **(A)** A total of 113 kinds of prediction models were evaluated via 10-fold cross-validation framework, and the AUC value of each model across all validation datasets was further calculated. **(B, C)** Selection of optimal λ for the Lasso model based on 10-fold cross-validation and generation of Lasso coefficients of the predicted CRGs in GSE5281. **(D)** The specific coefficients of 9 CRGs finally obtained by the optimal lambda value in the Lasso model. **(E–J)** ROC curves depict the performances of riskScore, age, and gender in predicting the initiation of AD in six representative validation datasets.

To further screen for characteristic CR regulators associated with AD, these differentially expressed 201 CRGs in single-cell dataset were first fitted into our machine learning-based integrative model to establish a consensus CRD signature. A total of 110 kinds of prediction models in the GSE63060 were performed via 10-fold cross-validation, and the AUC value of each model across all validation datasets, including the GSE140829, GSE33000, GSE36980, and GSE122063, was calculated. It is worth noting that the combination of RF+Lasso was identified as the optimal model with the highest average AUC value (0.753). Furthermore, this combined model also exhibited a relatively high AUC value in all validation datasets (**Figure 8A**). Based on the expression landscapes of 201 differentially expressed CRGs, the Boruta algorithm identified 27 important CRGs in GSE5281. Subsequently, these 27 CRGs were fitted into the Lasso model based on their expression data, and the optimal lambda value of 0.0222 was proved to achieve the minimum partial likelihood deviance based on the 10-fold cross-validation (**Figures 8B, C**). Finally, the Lasso-based machine learning model identified a set of 9 characteristic CRD signatures with non-zero coefficients (**Figure 8D**).

Next, utilizing the expression of 9 characteristic CRGs weighted by their coefficients in the Lasso model, a riskScore was calculated for each patient. Six external datasets were employed to compare the diagnostic efficacy of riskScore with other clinical variables (age and gender) in predicting AD progression. It was found that the riskScore displayed notably superior accuracy to other clinical characteristics, including age and gender in GSE5281, GSE33000, GSE122063 and GSE140829 (GSE5281: AUC=0.960, GSE28146: AUC=0.727, GSE33000: AUC=0.917, GSE122063: AUC=0.781, GSE140829: AUC=0.769, and GSE36890: AUC=0.718) (**Figures 8E–J**). These results led us to infer that the 9 CRGs-based riskScore may provide novel insights into the initial diagnosis of AD.

### Validation in pan-cancer cohorts and cortical neurons

**Figure 9:**
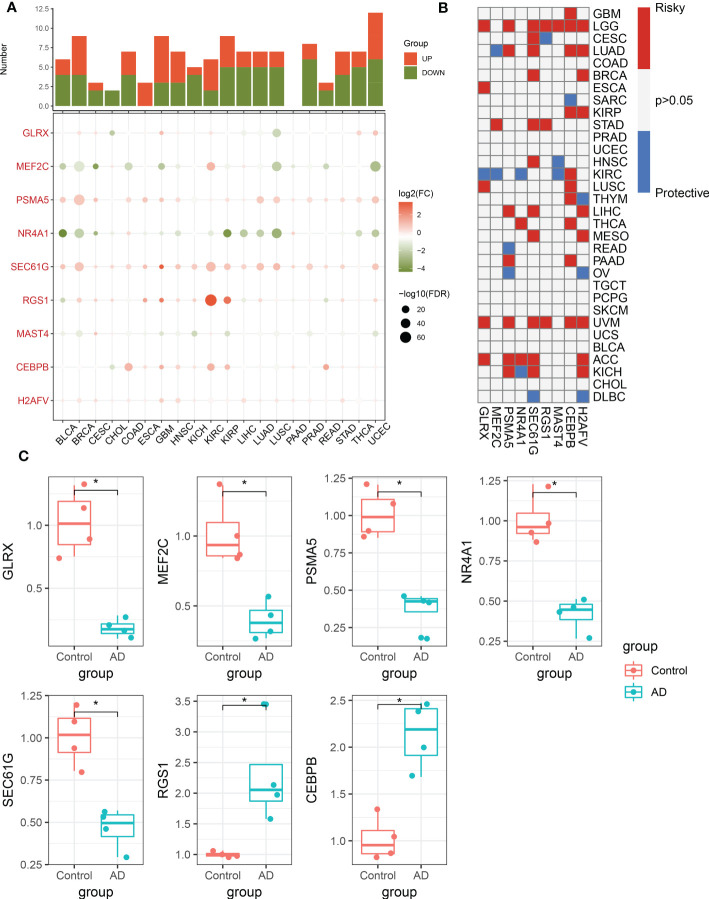
Pan-cancer and experimental validation of CRD signatures. **(A)** Histogram (upper panel) exhibits the number of DEGs that are up-regulated or down-regulated, and the heatmap depicts the fold change and FDR of 9 CRD signatures in each cancer. Red and green colors represent up-regulated and down-regulated CRD signatures, respectively. **(B)** Heatmap depicts the correlation between 9 CRD signatures and cancer patient survival. Red color corresponds to a greater expression of a CRD signature associated with poorer survival, while the blue color corresponds to a better survival association. Only p-values less than 0.05 were displayed. **(C)** Bar plots exhibiting the expressional differences in GLRX, MEF2C, PSMA5, NR4A1, SEC61G, RGS1, and CEBPB between control and AD groups. *p < 0.05.

To further validate the performance of the characteristic CRGs constituting the riskScore, we next explore the expression levels of these CRGs between the 20 cancer types and the corresponding adjacent normal tissues. The results demonstrated that MEF2C, NR4A1, and MAST4 were significantly downregulated in most of the tumors, while almost all tumors notably expressed PSMA5, SEC61G, RGS1, CEBPB, and H2AFV (**Figure 9A**). In addition, we aimed to illustrate the correlation between these 9 characteristic CRGs and the prognosis of 33 types of cancer. It was found that all of these genes displayed a considerable correlation with overall survival in at least three cancer types. In particular, H2AFV, CEBPB, SEC61G, GLRX, and PSMA5 exhibited dramatically worse overall survival in multiple cancers, which was consistent with their high expression in a variety of cancer tissues (**Figure 9B**). Additionally, RT-PCR analysis revealed that the expression levels of GLRX, MEF2C, PSMA5, NR4A1, and SEC61G were notably decreased in AD cortical neurons, while RGS1 and CEBPB genes were significantly greater in AD group relative to control sample (**Figure 9C**).

## Discussion

The current investigation found that the CR activity of B and T cells in individual AD patients differed significantly from the other immune cell types, lending credence to the idea that AD development is frequently accompanied with d less-robust circadian rhythms. B and T cells were shown to be involved in the immunological responses to AD and are strongly associated to the progression of AD (31–33), while the potential mechanisms have not been thoroughly explored. We first revealed the differences in the molecular properties of distinct levels of CR in B- and T-cell subtypes. Accordingly, we further evaluated the intercellular contacts between CRD-associated immune cell subtypes and AD cells at the single-cell level. This novel perspective allows us to comprehend how CRD in the cellular components of different AD immune microenvironments affects the outcome of individual AD patients.

Second, previous research reported that the progression of AD is inevitable and that infiltrating B cells in the brain parenchyma may lead to immunoglobulin deposition around Aβ plaques, thus favoring this process (31). Correspondingly, we found the CRscore was markedly higher in the B cells of patients with AD. It is worth noting that a total of four B cell subtypes in AD patients were observed in this study, all of which exhibited varying degrees of CR activity. In addition, we found that B cells with high-CR seemed to involve OXPHOS activity, together with microenvironment metabolism involving ascorbate, glycosphingolipid biosynthesis, tryptophan, β-Alanine, glycerophospholipid, pentose phosphate pathway, and glutamate, which play a critical role in promoting mitochondrial turnover and sustaining higher energetic activity, thereby facilitating the maintenance of mitochondria quality control in neurons (34, 35). However, low-CR B cells were mainly enriched in immune responses and calcium ion transport, accompanied by up-regulated pro-inflammatory genes and chemokine receptors. Furthermore, cellphone analysis exhibited that most ligand-receptor pairs, including BST2_LILRA4, CCL4_CNR2, GRN_TNFRSF1A, GRN_TNFRSFA1B, HLA-F_LILRB1, LGALS9_CD44, LGALS9_SLC1A5, and F10_TNFRSF10A communicated frequently in low-CR B cells. The increased chemokine CCL4 and GRN mutations were found to closely associated with AD pathology (36, 37), suggesting a poor prognosis in AD patients. Additionally, the pseudotime analysis demonstrated that the CRD is involved in the transformation of naive B cells into GC B cells, thus resulting in the progression of AD. Our results on the molecular differences in B-cell subtypes at different CR activity may illustrate, at least in part, the heterogeneity of AD, emphasizing the significance of additional research into the roles and tracer systems of CRD-based B-cell subtypes.

As the primary immune cells in the adaptive immune system, T cells are frequently dysfunctional in Alzheimer’s disease (AD) and are involved in AD pathology via contributing to neuroinflammation (38). It is demonstrated that AD patients and mouse models exhibit a greater number of CD4^+^ and CD8^+^ T cells relative to normal individuals, indicating an activated immune response (39, 40). Meanwhile, the heterogeneity of T cells in AD reveals patient responsiveness to treatment and influences prognosis (41–43). To date, few studies have thoroughly explored the specific T cell subtypes in the AD immune microenvironment, as well as the potential role of CRD in T cells. In our study, we consistently found a higher proportion of CD4^+^ and CD8^+^ T cells. Further subtype analysis revealed three subgroups in CD4^+^ T cells, including CD4_C01_ANXA1, CD4_C02_CCR7, and CD4_C03_CCL5. While we could classify CD8^+^ T cells into CD8_C01_CCR7, CD8_C02_GNLY, CD8_C03_GZMK, and CD8_C04_SLC4A10, all of which displayed distinct expression patterns. Interestingly, we identified that the CD4^+^ and CD8^+^ T cells in the low-CR group all had an elevated expression of antigen presentation and chemokine receptors. In addition, classical pathogenic pathways analysis indicated the involvement of low-CR CD4^+^ and CD8^+^ T cells in NFkB, TNFα, p53, MAPK, and hypoxia associated signaling pathways, while the high-CR group were mainly regulated by metabolic-related processes. It is well known that these activated pathways could promote AD development (44–48). Therefore, these results explain, at least in part, that low-CR activity CD4^+^ and CD8^+^ T cells can collectively contribute to the pathology of AD by mediating the activation of these pathogenic pathways. Further trajectory analysis indicated effector CD4^+^ and CD8^+^ T cells might be generated by the naive CD4^+^ and CD8^+^ T cells, which might be driven by CRD. It is worth noting that compared to the high-CR group, the expression of proinflammatory factors and MMPs, such as CXCL12, IL6, MMP9, and MMP11, was significantly elevated in CD8^+^ T cells with low-CR, while low-CR CD4^+^ T cells exhibited a higher expression of protein export factors. Additionally, CRD-mediated CD4^+^ and CD8^+^ T cells also showed a distinct expression landscape of metabolism-related genes, suggesting the CRD-induced T-cell dysfunction may be subtype-specific.

To further estimate cell-specific gene regulatory networks associated with CRD, we conducted an analysis of TFs at the single-cell level. In general, CRD-based B cells, CD4^+^ T cells, and CD8^+^ T cells all manifest distinct TFs characteristics. For B cells, most TF gene signatures were markedly enriched in low-CR group relative to high-CR group, such as NF-kB, FOS, ATF5, and RELB. Previous studies have reported the relationship between circadian rhythm-related genes and the expression of NF-kB, FOS, ATF5, and RELB (49–52), suggesting the role of CRD in the regulation of B cells. Moreover, for CD4^+^ and CD8^+^ T cells, we also observed distinct TF features of CRD-mediated cell subtypes. Overall, CRD-mediated cell subgroups may form distinct TF regulatory networks to reconstruct and reprogram the immune microenvironment of AD. Furthermore, cell-cell communication analysis revealed that these CRD-mediated immune cells exhibited a variety of ligand-receptor pairs, indicating that the dysregulation of AD microenvironment might be partially influenced by CRD. More effort is urgently needed to investigate the key TFs and ligand-receptor pairs that regulate the CRD-based immune cells and determine their function.

Considering the intricate intrinsic patterns of CRD in the immune microenvironment of AD, we further summarized the relationships of CRscore with pathology and immune response from the public bulk RNA-seq AD cohorts. In addition, only a few patients responded favorably to the treatment of AD (53–55), and the accuracy of biomarkers or models in early diagnosis needs to be further improved. Our current results suggest that lower CR activity is notably associated with AD-related pathological markers and pathogenic pathways, which have been shown to predict AD progression. Meanwhile, CRscore at different levels also exhibited markedly distinct immune cell infiltrating, immunomodulator expression, and immuneScore, suggesting that AD patients with different CR activity have distinct immune therapy responses. In view of this, we aim to develop an integrative pipeline to construct a characteristic CRD signature on the basis of the expression profiles of these differentially expressed CRGs. A total of 110 kinds of machine learning models were fitted to the training dataset via 10-fold cross-validation, and the subsequent validations in four independent test datasets demonstrated that the combination of RF+Lasso was identified as the optimal model. The benefit of integrative procedures is that they can be utilized to fit a model with consistent performance in AD diagnosis using various machine learning approaches and their combinations. The combination of algorithms could further eliminate the dimensionality of variables and makes the model more straightforward and plausible. The constructed riskScore based on nine characteristic CRGs demonstrated that the riskScore could maintain higher accuracy and more stable performance than age and gender on other independent validation datasets, suggesting that the riskScore has great potential for clinical applications in AD diagnosis.

Finally, we performed external validation in pan-cancer cohorts and expression experiments in cortical neurons to elucidate the specific roles of these characteristic CRGs in disease progression. Of note, the results suggested that all characteristic CRGs were strongly associated with overall survival in multiple cancer types, which was consistent with the findings that CRD is one of the causes contributing to the development of various cancers (56–58). RT-PCR analysis of cortical neurons revealed that the initiation of AD was often accompanied by a dysregulation of these characteristic CRGs, indicating that the deterioration and progression of diseases might be partially determined by CRD.

Some limitations need to be elucidated in our study. First, since our research was primarily based on retrospective studies, the robustness of our predictive model was limited. The findings of our research should be verified in a prospective cohort of AD patients. Second, additional clinical factors should be enrolled in the prediction model to improve accuracy and diagnostic performance. In addition, further experiments are needed to investigate the potential functional mechanisms of CRD in regulating AD.

## Conclusion

We, for the first time, revealed CRD-based cell subtypes in AD microenvironment at single-cell level. Based on a variety of bioinformatics and machine leaning approaches, we comprehensively illustrate the expression landscapes of circadian‐related genes and established a reliable and powerful signature for AD diagnosis. Our findings broaden the understanding of biological function heterogeneity and CRD-induced remodeling of the AD immune microenvironment. Such knowledge is critical for better developing and improving responsiveness to AD treatment, as well as for guiding the development of individualized therapies for AD patients.

## Data availability statement

The original contributions presented in the study are included in the article/**Supplementary Material**. Further inquiries can be directed to the corresponding author.

## Ethics statement

The animal study was reviewed and approved by the Institutional Animal Care and Use Committee of Fujian Medical University.

## Author contributions

MY and HH conceived and designed the study. HH, YY, LW, ZG, and LY collected the initial data and conducted the analysis. HH and YY accomplished the original manuscript. WO-Y and LW performed literature searches. HH carried out the RT-PCR analysis. HH, MY, and YY reviewed and edited the paper. All authors contributed to the article and approved the submitted version.
