# Supplementary material for: Combined analysis of single-cell and bulk RNA sequencing reveals the expression patterns of circadian rhythm disruption in the immune microenvironment of Alzheimer’s disease
Source: Front Immunol. 2023 May 12;14:1182307. doi: 10.3389/fimmu.2023.1182307 (PMC10213546; doi:10.3389/fimmu.2023.1182307)
Supplement: Supplementary file 1 [file DataSheet_1.docx]

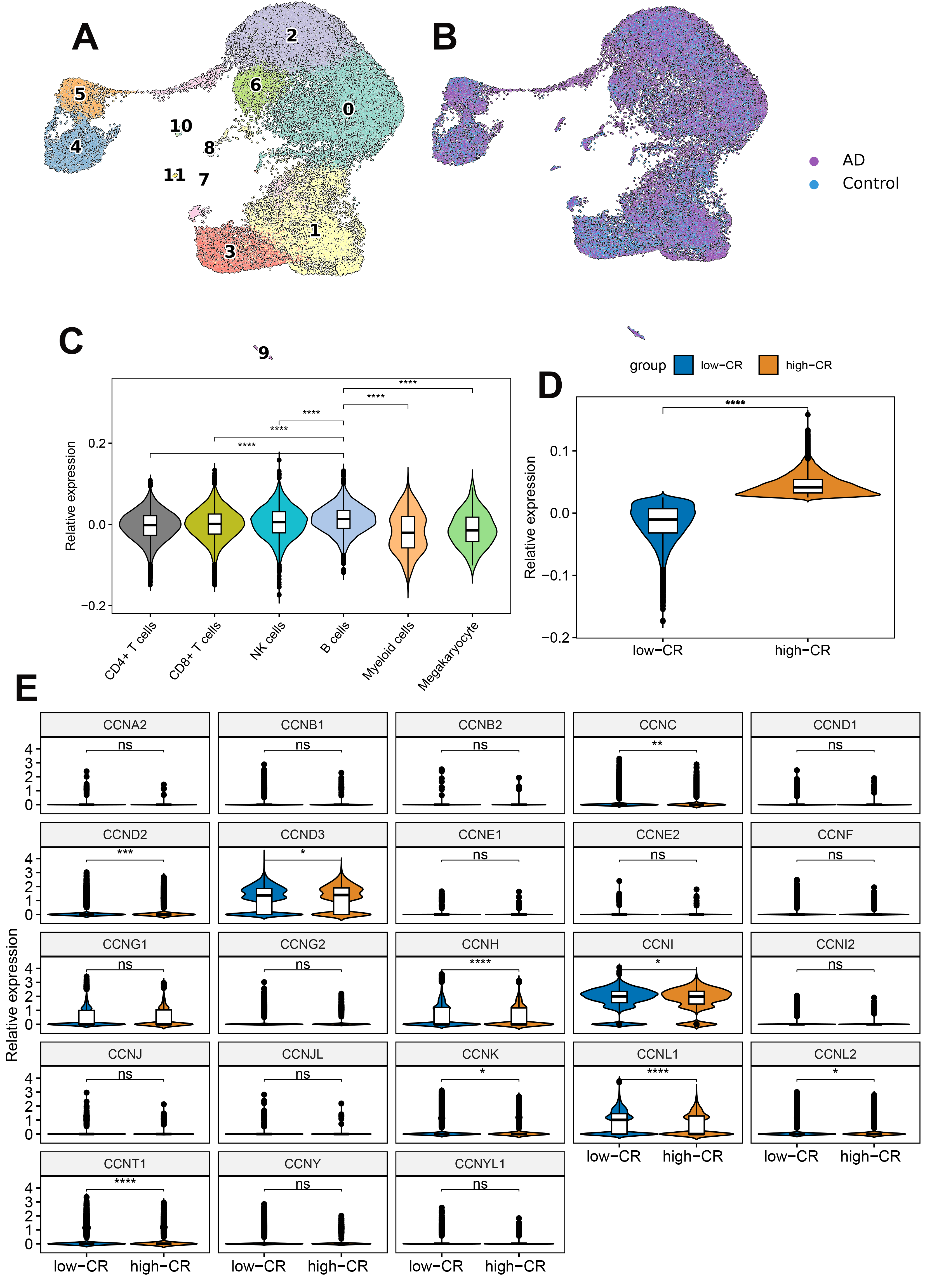


**Figure S1 Characteristic of CRD at the single-cell level.** (A,B) Cell clusters (A) group distribution (B) via the UMAP plot of 36725 cells. (C) Violin plots depicts the differences in CRscore among various cell types. (D) The CRscore of low- and high-CR patients are compared. (E) Violin plots depicts the expression landscapes of cyclin genes between low- and high-CR group.


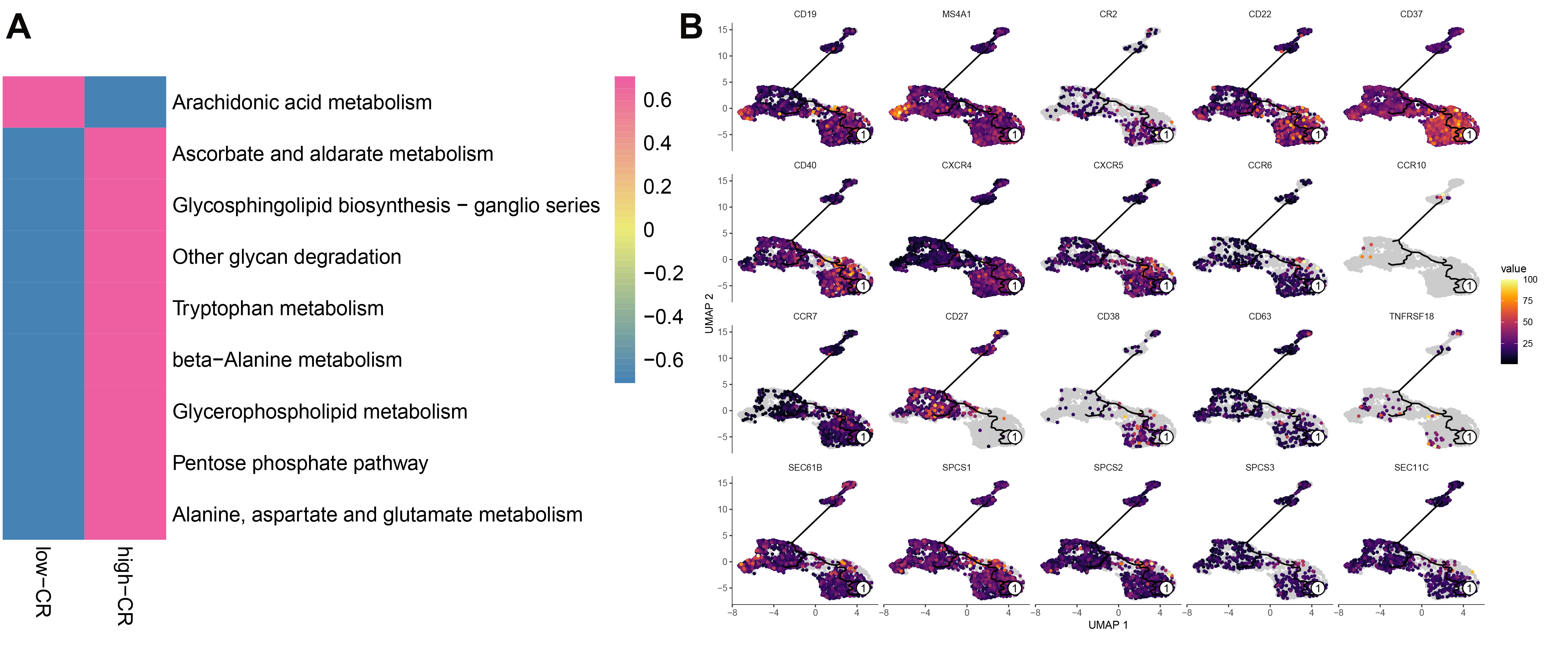


**Figure S2 CRD modified the features of B cells.** (A) Heatmap exhibits significantly different metabolic pathways between low- and high-CR B cells in AD patients. (B) Visualization of the expression of surface markers, chemokine receptor, and protein export related genes during the differentiation process of B cells.


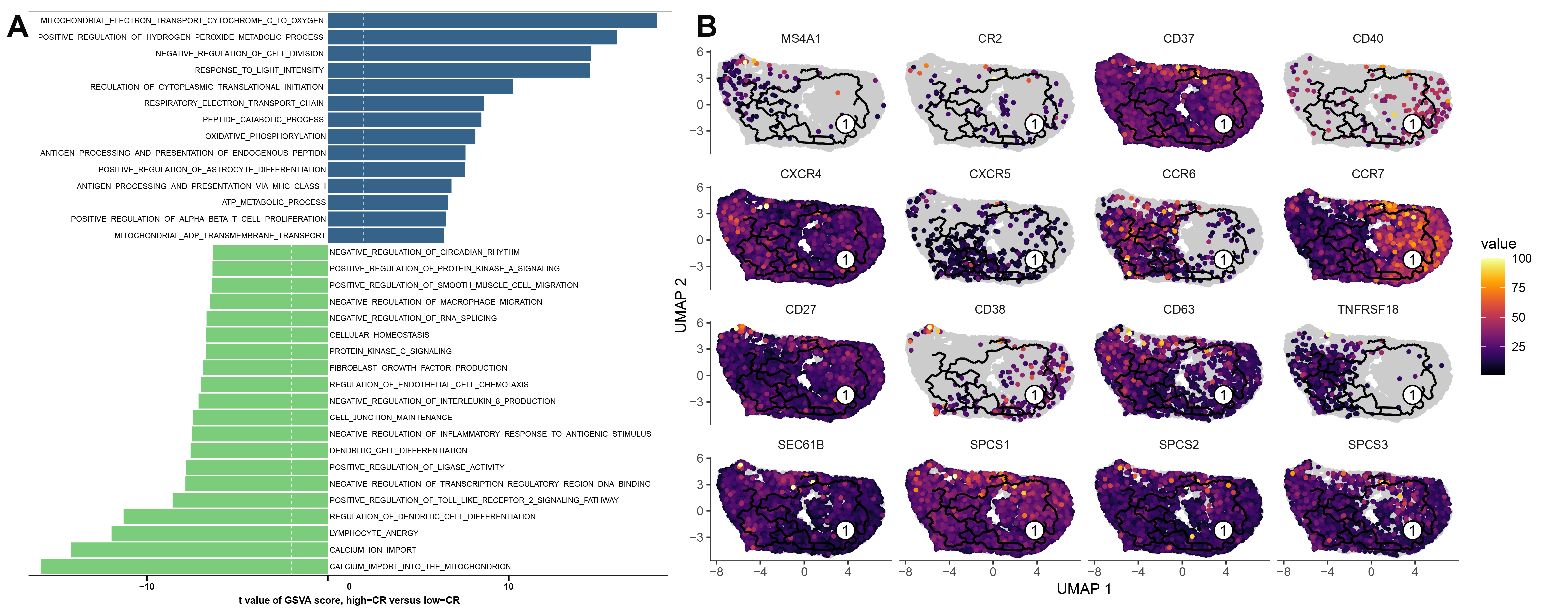


**Figure S3 Molecular characteristics in CRD-based CD4^+^ T cells.** (A) GSVA depicts the abundant biological functions between low- and high-CRscore CD4^+^ T cells in AD patients. Higher t value represents more enriched biological functions. (B) Visualization of the expression of surface markers, chemokine receptor, and protein export related genes during the differentiation process of CD4^+^ T cells.


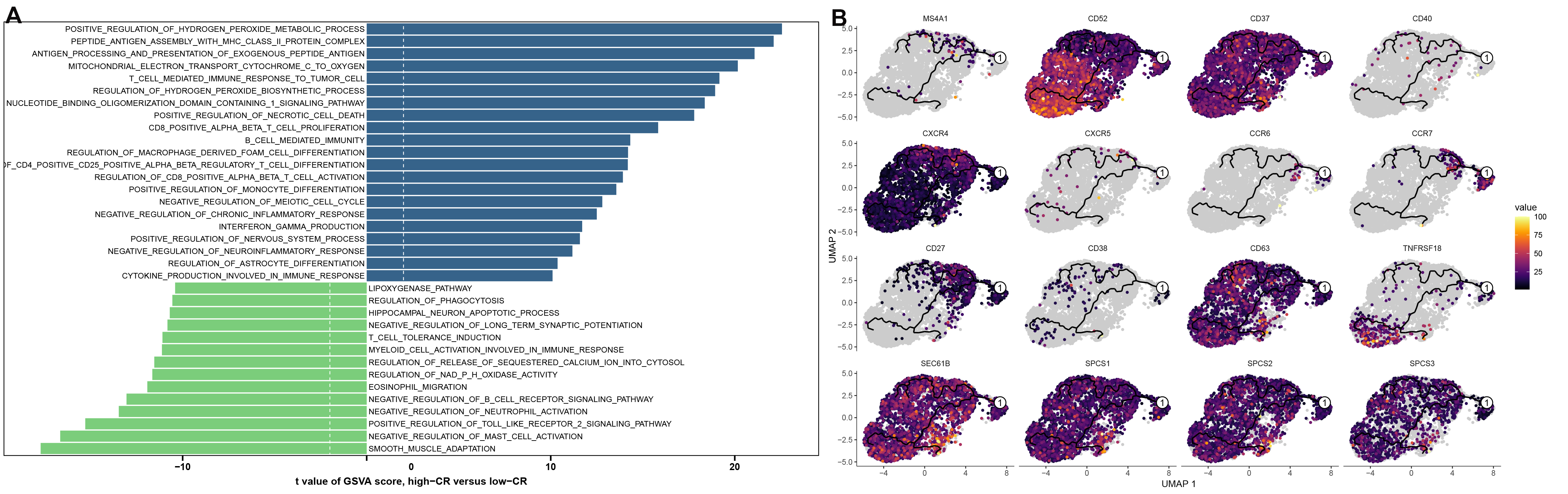


**Figure S4 Molecular characteristics in CRD-based CD8^+^ T cells.** (A) GSVA depicts the abundant biological functions between low- and high-CRscore CD8^+^ T cells in AD patients. Higher t value represents more enriched biological functions. (B) Visualization of the expression of surface markers, chemokine receptor, and protein export related genes during the differentiation process of CD8^+^ T cells.


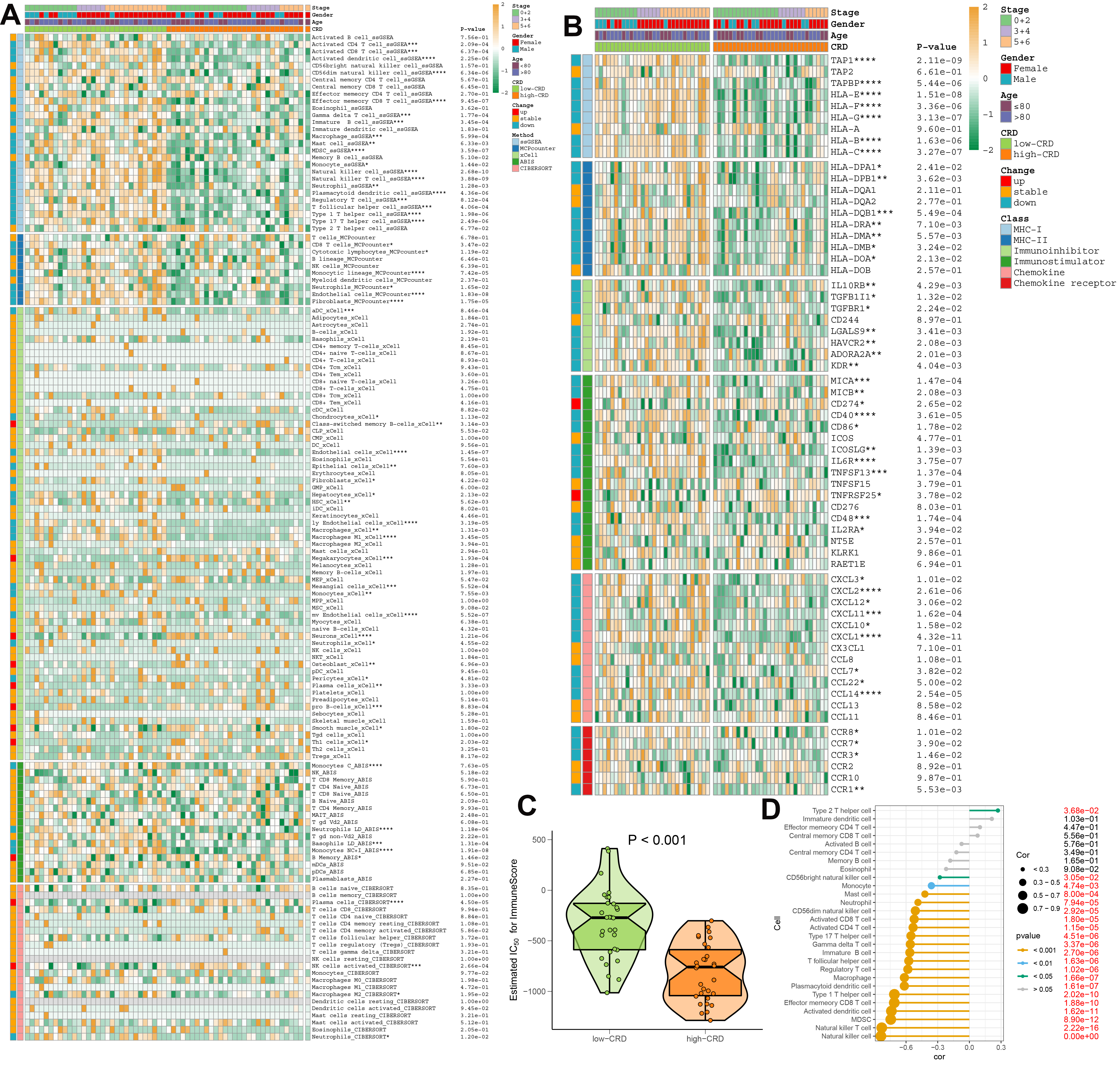


**Figure S5 The immunological characteristics of AD patients with low- and high-CRscore in GSE5281**. (A) Heatmap exhibits the expression landscapes of infiltrated immune cells between AD patients with low- and high-CR based on the ssGSEA, MCPcounter, xCell, ABIS, and ESTIMATE algorithms. **p* < 0.05, ***p* < 0.01, ****p* < 0.001, *****p* < 0.0001. (B) Heatmap exhibits the expression landscapes of immunoregulatory genes between AD patients with low- and high-CR. **p* < 0.05, ***p* < 0.01, ****p* < 0.001, *****p* < 0.0001. (C) The immunological score of low- and high-CR patients are compared. (D) Lollipop plots depict the correlation between CRscore and 28 immune cell subpopulations.
