# Supplementary material for: Combined analysis of single-cell and bulk RNA sequencing reveals the expression patterns of circadian rhythm disruption in the immune microenvironment of Alzheimer’s disease
Source: Front Immunol. 2023 May 12;14:1182307. doi: 10.3389/fimmu.2023.1182307 (PMC10213546; doi:10.3389/fimmu.2023.1182307)
Supplement: Supplementary file 2 [file Table_1.docx]

**Table S1 The information of public datasets containing microarray data in the study**

| GO accession | Platform | Samples | Sample source |
| --- | --- | --- | --- |
|  |  | Control AD |  |
| GSE106241  GSE48350  GSE84422  GSE5281  GSE28146  GSE122063  GSE33000  GSE140829  GSE63060  GSE36980 | GPL24170  GPL570  GPL570  GPL570  GPL570  GPL16699  GPL4372  GPL15988  GPL6947  GPL6244 | 0 60  173 80  28 7  74 87  8 22  44 56  157 310  249 204  104 145  47 32 | Brain tissues  Brain tissues  Brain tissues  Brain tissues  Brain tissues  Brain tissues  Brain tissues  Peripheral blood  Peripheral blood  Brain tissues |
